# Ageing Safely in the Digital Era: A New Unobtrusive Activity Monitoring Framework Leveraging on Daily Interactions with Hand-Operated Appliances

**DOI:** 10.3390/s22041322

**Published:** 2022-02-09

**Authors:** Hafsa Bousbiat, Gerhard Leitner, Wilfried Elmenreich

**Affiliations:** 1 DECIDE Graduate School, Alpen Adria Universitaet Klagenfurt, 9020 Klagenfurt, Austria; hafsa.bousbiat@aau.at (H.B.); wilfried.elmenreich@aau.at (W.E.); 2 Institute for Informatics Systems, Alpen Adria Universitaet Klagenfurt, 9020 Klagenfurt, Austria; 3 Institute for Networked and Embedded Systems, Alpen Adria Universitaet Klagenfurt, 9020 Klagenfurt, Austria

**Keywords:** active and assisted living, abnormal behaviour detection, smart metering technology, non-intrusive load monitoring

## Abstract

Supporting the elderly to maintain their independence, safety, and well-being through Active Assisted Living (AAL) technologies, is gaining increasing momentum. Recently, Non-intrusive Load Monitoring (NILM) approaches have become the focus of these technologies due to their non-intrusiveness and reduced price. Whilst some research has been carried out in this respect; it still is challenging to design systems considering the heterogeneity and complexity of daily routines. Furthermore, scholars gave little attention to evaluating recent deep NILM models in AAL applications. We suggest a new interactive framework for activity monitoring based on custom user-profiles and deep NILM models to address these gaps. During evaluation, we consider four different deep NILM models. The proposed contribution is further assessed on two households from the REFIT dataset for a period of one year, including the influence of NILM on activity monitoring. To the best of our knowledge, the current study is the first to quantify the error propagated by a NILM model on the performance of an AAL solution. The results achieved are promising, particularly when considering the UNET-NILM model, a multi-task convolutional neural network for load disaggregation, that revealed a deterioration of only 10% in the f1-measure of the framework’s overall performance.

## 1. Introduction

The growing rate of the elderly population has been a central topic in the last decade and will remain relevant for the coming decades; the lifespan of people has become longer than it has ever been. Such development will undoubtedly impact economic and societal systems, including healthcare services. In Europe, for example, the ratio between people of working age and those aged above 65 years was 4:1 in 2001 [1]. It is estimated that by 2050 there will be fewer than two persons of working age for each elderly person [1]. Not only is the ratio estimated to change significantly, but also the number and severity of illnesses. The EU accounted for more than 9.1 million cases of individuals older than 60 years with dementia in 2018, which was only 5.9 million in 2000 [2]. Thus, if the age-explicit predominance of dementia increases at the same speed, the growing ageing population will translate into an increased number of dementia cases. It is thus expected that by the year 2050, there will be approximately one million new cases of dementia every year that need health support [3]. Professional care-giving institutions and practitioners would quickly be overwhelmed with the growing number of patients at different stages in their disease. Aware of these challenges, health care professionals are becoming increasingly open to using technology that supports them in performing their work [4].

A new set of technologies, titled Active and Assisted Living (AAL) technologies, have emerged in response to these challenges. They refer to all products and services that are designed to support individuals in retaining or enhancing their independence and well-being [5,6]. The main goal of this collection of diverse technologies is to offer an automatic and accurate alternative to classical data gathering techniques (e.g., through recurrent questionnaires) used for further decision making and thus assist healthcare professionals in efficiently monitoring the elderly.

Existing AAL approaches rely on deploying several sensors inside the house to enable ageing-in-place. Nevertheless, adopting this approach appears intrusive and, in some cases, expensive for older adults. The intrusiveness is generally related to solutions that consider sensors intervening in the daily activities or violating privacy constraints. The subjects are thus less accepting toward these solutions [6,7]. On the other hand, solutions based on background sensors may appear to be a viable alternative. However, the costs of their purchase, installation, and maintenance remain a major obstacle. The Casa Vecchia [8] project conducted in the rural areas of Austria highlighted this issue. The authors stressed the economic aspect of AAL solutions based on smart technologies. According to the authors, the price of basic solutions starts from 150€ and could rise to 90,000€ for the most sophisticated solutions without considering installation and maintenance costs. The latter also contributes to the high intrusiveness of such kinds of solutions.

A promising sub-field of AAL relies on the indirect monitoring of daily activities through energy data generated by the explicit usage of electric devices [5]. These daily activities are direct indicators of daily routines and well-being. Moreover, they could also assist in recognising the beginning of cognitive impairments that start with difficulties in performing complex instrumental tasks and continue to a total loss of the ability to perform basic daily tasks [9]. Mainly, difficulties in performing daily activities translate into deviations in the usage patterns of hand-operated appliances, reflected in the recorded energy data. The described approaches benefit from the extensive deployment campaigns of smart-meter worldwide. These campaigns provide an already available infrastructure that can be–compared to sensors that would have to be installed additionally–easily used whenever needed. These approaches also benefit from the non-intrusiveness of smart-metering technology. The occupants will not perceive any changes since no additional installation or maintenance tasks are necessary. Instead, occupants conduct their regular routines with no differences. Nonetheless, these approaches can not be attainable without Non-intrusive Load Monitoring (NILM), a set of techniques interacting with the smart meter to identify the power consumption of different appliances.

A primary challenge in monitoring daily routines (and identifying significant deviations) is to design systems considering the heterogeneity and complexity of daily routines. Moreover, scholars gave little attention to evaluating state-of-the-art NILM models in AAL applications. We suggest a new framework for activity monitoring based on custom user-profiles and deep NILM models to address these gaps. Furthermore, we propose an interactive framework by exchanging feedback with external agents such as health care professionals or family members. The key objective of our work is to provide them with a tool that can take over basic monitoring means which currently require manual efforts (such as frequent phone calls or visits). This is particularly relevant for the case of rural areas and older relatives living alone. When the technology works appropriately, phone calls and personal visits can focus on social and relationship aspects rather than daily activities monitoring. The contributions of the presented work fall in two main points: (1) The proposition of a new activity monitoring framework based on the usage time of hand-operated appliances inferred from energy data, and (2) the discussion of two case studies of houses occupied by older adults from the REFIT dataset where we evaluate the whole proposed pipeline including NILM approaches and their influence on activity monitoring.

The remainder of the paper proceeds as follows: Section 2 gives a brief overview of existing work on activity monitoring using NILM. Section 3 describes the details of the proposed framework and its different modules. Section 4 presents the data used as well as the methodology adopted to evaluate our contribution where the results are illustrated in Section 5. Section 6 analyzes the obtained results and reveals the main findings of our work. Section 7 concludes by presenting the main limitations of the current work and suggestions for future enhancements.

## 2. Related Work

### 2.1. The Importance of Daily Activities

The set of habits and routines carried out by individuals for self-caring is formally referred to as Activities of Daily Living (ADLs) and Instrumental Activities of Daily Living (IADLs). ADLs include basic self-care tasks summarised in six categories: eating, bathing, dressing, toileting, mobility, and grooming [10]. On the other hand, IADLs refer to more complex tasks. These tasks include shopping, preparing meals, using the telephone or other communication devices, managing medications, doing laundry, housekeeping (cleaning, tidying up, removing trash and clutter, and folding clothes), and essential home maintenance [11]. Difficulties in accomplishing these tasks as well as abnormal deviations in the inhabitants’ behaviour are clear indicators of cognitive diseases in early stages [4]. Scientifically validated tools (e.g., SMAF [12] and the Lawton scale [13]) include, among others, measures indicating that observing the usage of home appliances provides insights about the regularity of a person’s activity. Moreover, recent works [9] demonstrated that the housekeeping-related activities component allows predicting the potential of having dementia for individuals with mild cognitive impairments (MCI) three years prior medical diagnosis.

### 2.2. Appliances Involved in Instrumental Daily Activities

The current manuscript focuses on a subset of IADLs highly correlated with the use of hand-operated electrical appliances [14] as illustrated in Table 1. In this respect, we are not primarily interested in the possible development of psychological problems but in identifying typical daily routines. It is possible to analyse IADLs using appliances’ usage directly derived from their energy consumption. Consequently, monitoring a more extensive set of activities would require identifying a larger group of appliances. However, it is worth mentioning that not all electrical appliances are good candidates for identifying daily routines and activities. The appliances considered should be manually operated, frequently used on a daily basis [7]. The *Kettle*, for example, was identified as a good candidate in several works [7] as it is used daily and several times during a single day. Nevertheless, some passive appliances could also provide evidence about the normality of behaviour. For example, the fridge has a periodic behaviour that does not require human intervention and is, therefore, less informative in the scope of daily activities recognition. However, it is a good indicator of anomalies in the behaviour, as previously demonstrated in [15]. Moreover, when combined with the usage of a TV, it provides a good indicator for sleep disorder [6]. In summary, the activity monitoring of the elderly using energy data is concerned with two main problems, the identification of appliance usage and the modelling of daily routines.

### 2.3. Non-Intrusive Load Monitoring (NILM)

Two viable strategies are recognised to quantify the power usage of appliances present inside a household. The first strategy, referred to as Intrusive Load Monitoring (ILM), requires the connection of each appliance to a metering point. It follows from this definition that this alternative is expensive and complex to maintain. Furthermore, subjects could consider it to be intrusive to their daily activities. The second strategy, referred to as Non-Intrusive Load Monitoring (NILM) or load disaggregation, follows the approach of decomposing the total power consumption of the household into the different contributions of each appliance [16] using only a single metering point, the smart electrical meter. It provides a cost-efficient solution that can be used massively and discreetly inside the household. Therefore, it is considered to be the optimal means to identify the usage of appliances inside a home. The concept of NILM was first introduced by George W. Hart [17] 30 years ago. However, it only gained significant interest from the research community in recent years due to the update of the electrical grid around the globe. The goal of NILM approaches is mainly to model the patterns of electrical signatures related to different appliances using advanced statistical models. In this matter, scholars distinguish between four types of appliances [17]: (1) On/off appliances such as lights, which represent appliances that have only two operational states and are either on or off, (2) multi-state appliances that operate according to a finite state machine such as a washing machine, (3) continuous consumption appliances which gather new devices that have an infinite number of states such as computers and laptops, and finally (4) background appliances that are turned on the whole time, such as routers. The first three categories of appliances are the most relevant ones in the scope of daily activities. Identifying their power consumption can be achieved using event-based or event-less approaches. The first set of approaches is interested in identifying ON/OFF events, while the second is more interested in directly estimating power consumption. Both methods are viable for IADL applications. Factorial Hidden Markov Models (FHMM) are among the first models that were extensively investigated in the literature to model different appliances’ signatures [18,19,20] where the observed variable represents a function of the aggregate power, and the hidden variables represent the states of appliances. However, these probabilistic models are not able to model all types of appliances, and they become computationally inefficient with the increasing number of states. Recently, deep models became a main research stream in the NILM scholarship [21] due to their ability to automatically learn which features to extract from a dataset and to generalise to new and unseen data [22,23,24,25,26,27,28,29,30,31]. Even more interestingly, recent literature demonstrated the superiority of these models upon existing probabilistic models on different datasets [32]. Among recently proposed approaches, multi-task deep NILM models [33] performing both power and state estimation of appliances demonstrated interesting enhancements. Deep NILM models seem to be a promising field for improving disaggregation performance. Nonetheless, the comparability problem in the deep NILM scholarship remains a persistent gap [34] due to the different evaluation setups used to validate these models.

### 2.4. Learning the Usage Patterns of Appliances

Zhang et al. [14] obtained interesting results for 14 types of ADLs through an approach that learns a personal model that is aware of the context of ADLs from appliance power consumption. The authors of [16] proposed comparing both active and reactive power and time of day with a pre-build database of signatures to identify concrete appliances. However, this approach requires human intervention, which does not satisfy an essential condition in the NILM requirements proposed by Zeifman [35]. Another weakness of the approach is the limited availability of a vast database containing signatures of appliances from different manufacturers. In a later work, Alcala et al. [7] proposed a double stochastic process (a Cox process) to encode the periodicity of usage patterns and stressed the importance of the kettle in such applications. In [6], the authors suggest a two-phase model: A learning phase–where a basic model learns the inhabitant’s routines and daily activities during which the system is unable to detect abnormality–and an operational phase where the system employs the learned model for reporting and analysing the detected activities. Despite the interesting suggestion, this approach does not consider variations in the behaviour that could occur after the training phase. More recently, in [4], the authors suggested using the indices of usage patterns to detect deviations in the behaviour relying on the Dempster–Shafer Theory. Projects such as city4age [36,37] that was initiated in Europe highlighted the importance of using load disaggregation in smart homes for monitoring the elderly population but also discussed the reliability issue on such a system. For example, turning on the TV does not forcibly mean that the subject is watching TV. It could be the case that the subject turned on the TV and left the room. Such cases would lead to a high number of misinterpretations and, therefore, wrong diagnoses. Motion sensors could be combined with NILM to overcome this issue. Table 2 provides a summary of ADLs monitoring techniques based on NILM. An even more detailed review of AAL approaches based on NILM can be found in [5,38].

Although previously presented contributions showed promising results, a main missing point is the availability of annotated data to evaluate these approaches. Generally, the authors propose to evaluate in labs, simulating real homes (see Table 2). The only work using real energy data, from the HES [39] dataset, was presented in [4], which is also not publicly available. To the best of the authors’ knowledge, none of the previous work was designed to provide adaptive monitoring with interactive and interpretable feedback to external agents. Moreover, scholars in the field gave little attention to evaluating the influence of NILM on the proposed approaches. Most of the existing contributions thus suppose a perfect disaggregation of the total power consumption that is so far not reached in the NILM scholarship.

### 2.5. Active and Assisted Living and Non-Intrusive Load Monitoring

Active and Assisted Living (formerly Ambient Assisted Living) (AAL) aims to help the ageing population maintain autonomy and well-being. Achieving this goal is generally linked to tracking and monitoring daily routines and activities that is approached using two different techniques: (1) Direct techniques based on Wireless Sensor Networks (WSNs) ranging from lightweight systems to more sophisticated systems incorporating cameras and motion sensors [7], and (2) indirect techniques based on the observation of daily routines of the occupants. While the first set of approaches provides accurate and precise information, they are less accepted by subjects due to their intrusiveness leading–besides other acceptance issues–to privacy concerns. Another issue with this set of techniques is the complexity of their maintenance and the investment cost they require [6,7]. Furthermore, the high accuracy they provide is not required in all cases but rather only in severe ones where persons have chronicle diseases or are in a critical health situation [4]. The second set of approaches are comparatively less intrusive and rely on the analysis of daily activities of the inhabitant and the identification of abnormal patterns in daily routines that health care professionals consider as good indicators of well-being [6].

The availability of smart meters at a large scale and considerable low cost makes NILM an appealing solution for diverse tasks, such as energy-saving [40], but also for ADL recognition and abnormal behaviour detection potentially offered at a small additional cost from energy retailers [7]. This alternative provides an unobtrusive and economic monitoring possibility. It could support the health care system in the case of the elderly living alone, as this style of life (either voluntarily or involuntarily) is increasingly growing [1]. Despite the achievements of NILM in the recent past, the field remains challenging [4,21]. Therefore, monitoring ADLs based on the usage of appliances present in the household will certainly not provide as accurate information as solutions based on WSNs [4]. Using NILM in AAL applications would reduce the use of WSNs only for those extreme cases while still providing a certain level of safety and autonomy for people in standard cases. Thus, the premise of using NILM in AAL is instead to take advantage of the massive deployment of smart meters in the present era, their lower cost, and the non-intrusiveness of this approach [4] for monitoring the well-being of people in a good general condition as well as in the early stages of cognitive diseases.

## 3. Proposed Framework

Modelling and tracking human behaviour is a complex process that includes several aspects. In the scope of our contribution, we consider three main aspects. First, individuals have different routines and lifestyles, which highlights the importance of defining a custom daily profile, for each user, based on a pre-recorded historical energy consumption. This profile is the base for scoring a new day and deciding its normality. Demonstrative examples are different sleeping, eating, and waking up times. Second, human routines are subject to change. The profile must thus be adaptable to this dynamic character of human behaviour. In this regard, we differentiate between variations and deviations in the behaviour [16]. Variations are just new habits and routines adopted by the subjects. These new variations need to be included in the profile of the user. On the other hand, deviations are categorical changes in the routines translating into a potential abnormal pattern. An illustrative scenario for this case is cooking breakfast. A change in the breakfast time for several consequent days is considered a variation. However, the sudden cancellation of breakfast time would be a deviation that should be reported to an external agent for further decision-making. In this case, the role of the external agent is to confirm the abnormality of this cancellation through further investigations. Third, we use the feedback provided to update the profile in the case of misidentified anomalies. It would allow overcoming the non-availability of annotated energy datasets specifying anomalous events. The possibility of continuous integration of feedback from an external agent (e.g., health care professional or family member) would also help to improve future predictions and gather data labels in real-time.

**Figure 1:**
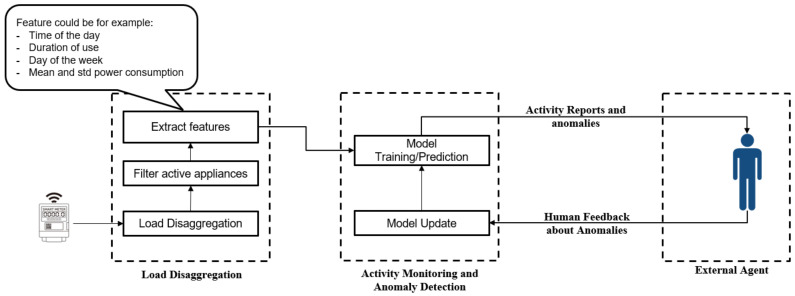
A general overview of the proposed framework.

The overall architecture of the proposed framework is illustrated in Figure 1. It is composed of three modules having independent tasks: a load disaggregation module, an activity monitoring module, as well as a feedback management module. In real implementations, the load disaggregation and activity monitoring modules can run locally within the household in the case of privacy-concerned users. In this regard, cost efficient implementations can be achieved through extending existing open source solutions. They can come as extra-modules to the OpenHAB (https://www.openhab.org/, accessed on 30 December 2021) platform, for example, as it already contains a load disaggregation module developed in a previous work of the authors [15]. Moreover, due to the sensitivity of the data, the communication with the external agent in real scenarios should be protected and secured using advanced smart grid security schemes (such as [41]), providing both low communication and computation costs.

The load disaggregation module is responsible for identifying features relative to the activation of different appliances. As an input, it uses the aggregate power of the whole household obtained with a single metering point, the smart electrical meter. The consumption of individual appliances is inferred afterward using load disaggregation techniques on an hourly basis. For active appliances, a summary of the current activation is extracted containing both contextual information (e.g., temporal information, weather information, other sensors information) and operational information (e.g., max power consumption). The operational information is related to the operational characteristics of the appliances. The goal from using these pieces of information is to confirm that the device used for monitoring an activity is not defective. For demonstration purposes, we only use the temporal data as contextual information in the remainder of the manuscript represented by: the start time of use, duration of use, the day of the week, and day of the month.

The activity monitoring module relies on the features created by the load disaggregation module to generate recurrent reports and anomalies that are transferred to an external agent to intervene whenever required. In regard to the first considered aspect (i.e., the heterogeneous routines of individuals), we suggest a two-phase functional mode for this module. The first phase is an observation phase in which the framework only enables module one and uses its outputs to build a custom consumption profile of the occupant. After this period, the framework becomes fully operational and can detect anomalies automatically.

The observation phase is based on passive monitoring where only daily reports are generated, and activity patterns are saved. For each day during this period, the daily routines are recorded for a set of pre-defined activities considering two levels. The first level is the activity level that is modeled using activity curves [42] taking into consideration two main aspects: the time of performing an activity and its duration. We argue that the combination of these two pieces of temporal information constitutes an interesting tool for monitoring the activities on a daily basis as they summarise the human interaction with the appliances represented by the ON event and the OFF event. Both features characterising an activity are inferred based on appliances involved in the activity where the mapping between the activities and appliances is pre-defined. The second level is the appliance level that can be represented, for example, using self-similarity measures [43]. The activity curves represent activity distributions along a given day where the probability distribution is defined as the normalised time an individual spends on an activity during a given period [42]. The duration of an activity during this specific period is the aggregation of the time spent on each related appliance. As previously mentioned, the custom user profile is constructed during this phase where the distribution of each activity is saved in a database of observations ∑. A summary representing the profile of the occupant can be extracted by aggregating the observations using the mean. It is worth mentioning that a more elaborated summary of the daily distributions would consider hierarchical aggregation based on different temporal levels. For example, an interesting approach would be to aggregate according to day time first (morning, afternoon, evening, night) or day of the week resulting in hierarchical activity curves.

During the second phase (i.e., the monitoring phase), the curve of each activity is calculated for the current day. At the end of the day, these curves are compared to the database of distributions recorded during the observation phase. The comparison measure used is the Jensen–Shannon Divergence (*JSD*) calculated as per Equation (1), which serves to measure the similarity between two or more distributions:

(1) $$JSD_{\pi_{1},\ldots ,\pi_{n}}\left(P_{1},\ldots ,P_{n}\right)=\sum \pi_{i}D(P_{i}||M);$$

(2) $$M=\sum \pi_{i}P_{i}\;,\;\sum \pi_{i}=1;$$

(3) $$D\left(P||M\right)=\sum P\left(x\right)log\left(\frac{P(x)}{M(x)}\right).$$

The $\pi_{i}$ are weights attributed to different observed days. In our case, they are used to give more importance to distances related to days where the external agent provided feedback. In particular, if $N_{1}$ is the number of days where the external agent provided feedback and $N_{2}$ is the number of days with no feedback, the $\pi_{i}$ is calculated as follows:

(4) $$\pi_{i}=\left\{\begin{array}{cc} \frac{1}{N_{1}\;+\;\beta N_{2}} & \mathrm{if}\;i\;\mathrm{contains}\;\mathrm{feedback}\\ \frac{\beta }{N_{1}\;+\;\beta N_{2}} & \;\mathrm{otherwise}\; \end{array}\right.,\;\mathrm{with}\;\beta \geq 2.$$

The comparison procedure relies on the Inter-quantile Rule (IQR) to decide about the normality of the current day. The distance between each observed day and the rest of the data is calculated to estimate the distribution of the distance in the case of the normal days. The IQR is calculated as the difference between the third $Q_{3}$ and first quantile $Q_{1}$. Under the assumption of normal distribution, the interval [Q1−1.5IQR, Q3+1.5IQR] contains 99.7% of the data. Thus, if the values of the distance of the current day fall inside this interval, it is considered a normal day with no deviations. On the other hand, if it falls outside of this interval, the current day is considered anomalous, and the activations of appliances related to the activity are further analysed.

**Figure 2:**
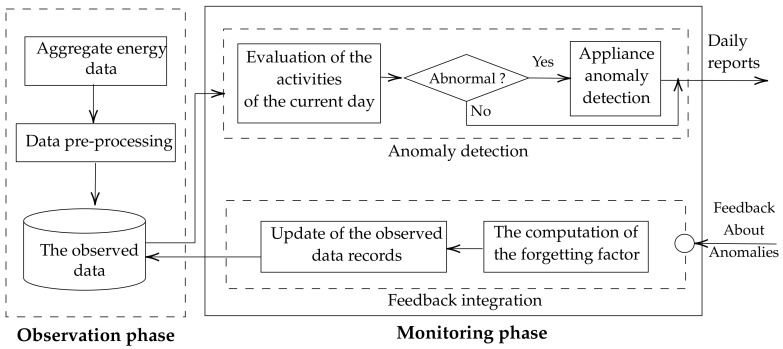
The monitoring and anomaly detection module.

Appliance anomaly detection is performed only in days that were classified as anomalous (see Figure 2) to provide a meaning-full interpretation of the causes behind the anomaly and provide detailed report about the events of the considered day. For this purpose, we suggest the use of a similarity-based approach [43] that relies on the calculation of self similarity matrices encoding the similarity (distance) of each observed activation to other historical activations. A threshold measure is used to decide if an activation is a variation (i.e., a small change in the routines) or a deviation (i.e., an anomaly) from the normal consumption pattern. The threshold, in this case, is also calculated using the inter-quantile range rule. More precisely, the threshold is directly deduced from the self-similarity matrices using the inter-quantile range rule.

Anomalous days result in notifying an external agent for further decision-making. The communication with the external agent is performed in an asynchronous mode. The notifications are sent to the external agent who has the choice to provide feedback or not to the framework. The framework continues thus to be functional even for days where the feedback was not received. In this case, it considers that the reports provided are correct and updates its historical data accordingly. This update allows the framework to consider recent patterns when evaluating a new day in order to take into consideration seasonal change in the routines. In the opposite case (i.e., if the feedback is received), if the agent finds the anomaly irrelevant, they can forward their decision to the framework. For interpretable feedback, we propose four classes of notifications: (1) Abnormal usage time which is related to an abnormal activation of an appliance during the day, (2) usage during the night, and (3) absence of activity during a day. Providing interpretable information about the anomalies would help to guide the external agent in establishing diagnosis.

The feedback provided by the external agent is integrated in the framework through an update of the database of historical consumption. The new activations are added to the existing database of historical events using a forgetting (vanishing) factor that favours the replacement of old and unlabelled entries. The replacement procedure is straightforward. It takes into consideration two main metrics: (1) how old the information is, (2) the distance to the rest of the historical data, and (3) external agent feedback. If all the days included in the observation database have feedback, the framework will choose the oldest most divergent day to be replaced. The replacement of a day *i* operates on two levels. First, the self-similarity matrices are updated by replacing the line *i* and row *i* with distance measures related to the current day. Second, the activity curve of the day *i* is tagged as having feedback and being replaced by the current day distribution.

## 4. Case Study

Aiming to evaluate the proposed framework in real scenarios, we present two case studies of the kettle’s usage relative to two different houses from a publicly available dataset. The two presented cases exhibited different usage patterns and were chosen to evaluate the proposed framework considering different routines. The kettle is used as a representative appliance for the cooking activity. The choice of this appliance is motivated by related literature arguing that its frequent daily usage is a good indicator of habits’ deviations [44,45,46]. In describing both use cases, we first detail the evaluation methodology while highlighting the differences in the data processing for each house. Second, we present a detailed description of the data obtained from both houses before and after pre-processing.

### 4.1. Methodology

**Figure 3:**
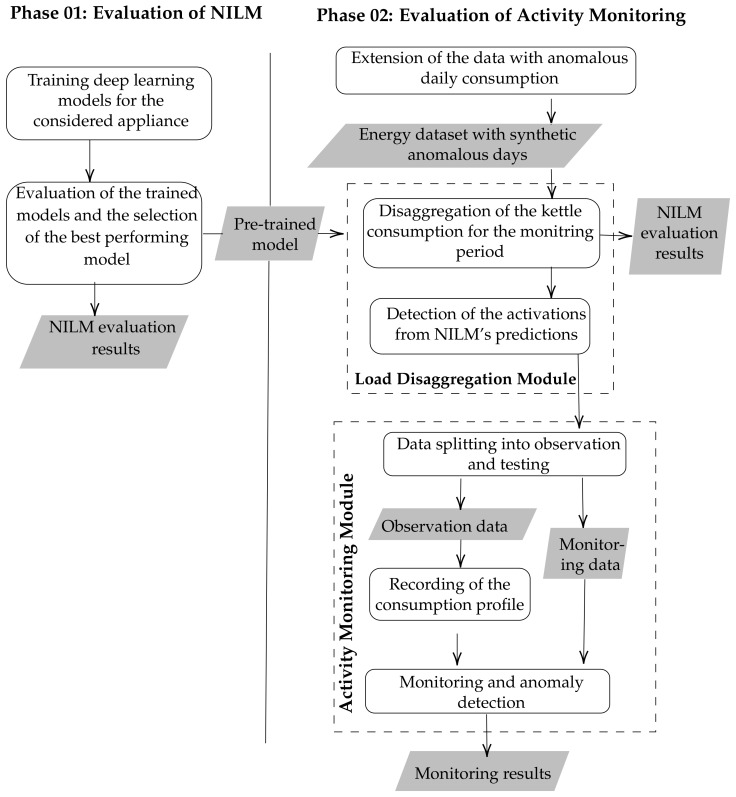
The evaluation methodology.

To evaluate our contribution, we suggest an evaluation procedure as described per Figure 3. It considers the different modules of the proposed framework while measuring the influence of earlier modules of the framework on the overall output. As the figure illustrates, the evaluation is organised in two phases.

First, we evaluate several disaggregation algorithms to identify the best-performing model in the case of the kettle. For this purpose, we consider six different disaggregation approaches: Combinatorial Optimisation (CO), Factorial Hidden Markov Models (FHMM), and four different deep models. The four deep models consist of two well-known NILM baselines, the Seq2Seq and Seq2Point models, and two recent models, the temporal-pooling model [47] as well as the UNET-NILM model [33]. The choice of the last two models is fueled by recent literature [21] suggesting their superiority. To train the deep models, we chose a training period of three months from house 4 and a testing period of one month from each house as illustrated in Table 3 at a sampling rate of 8 s where timesplit cross-validation with three folds was adopted. In particular, we evaluate the models in both seen and unseen scenarios to assess their performance in real setups. The seen scenario is a scenario where the energy data used for training and testing are from the same household but collected in different periods. In our case, it refers to the case where the models are trained and tested on data from household 4. On the other hand, the unseen scenario, refers to a testing scenario where the training and testing data are form different households. In our case, it refers to the case where the models are trained on household 4 and tested on data from household 11. The unseen scenario, in this case, is the most realistic as it is highly probable that the models will operate on houses that were never included in the training dataset.

During training, the data is split into 85% for training and 15% for validation. During testing, we provide special attention to classification performance metrics as ON events and usage time are more relevant in the case of activity monitoring. To construct the confusion matrix and derive the operational states from the power consumption, a threshold of 500 watts was used. All models were implemented using the PyTorch framework in a compatible format with NILMtk API [32]. We consider four different metrics to evaluate the generated predictions: The Mean Absolute Error (*MAE*), the *f*1-measure, the recall, and the precision, as demonstrated by the formulas below where *TP* represent the correctly identified ON states, *FN* represent undetected ON states, and *FP* represent the overestimated ON states:

(5) $$MAE=\sum_{0\leq i\leq T}\frac{|y_{i}-\hat{y_{i}}|}{T};$$

(6) $$f1-measure=2*\frac{Precision*Recall}{Precision+Recall};$$

(7) $$Precision=\frac{TP}{TP+FP};$$

(8) $$Recall=\frac{TP}{TP+FN}.$$

The first phase of the evaluation generates a set of pre-trained models that can be directly used to identify the kettle’s power consumption. As our purpose is to simulate a real scenario, the second phase of evaluation, which consists of evaluating the activity monitoring module, relies on the predictions generated by the best performing model during the first phase. Furthermore, due to the non-availability of annotated data, the authors suggest pre-processing the data extracted from both houses to generate an extended version of the data for both buildings, including labels relative to abnormal usage of the kettle. Three classes of the anomalous days are considered: (1) Divergent usage that corresponds to activations occurring at a non-usual time for the householder, (2) usage during the night, and (3) absence of usage during a day. When not available in the data, synthetic daily consumption of a class is inserted with a rate of 10% of the considered period’s length. Divergent usage patterns are generated by selecting a random day from the real dataset and altering the usage hour. On the other hand, the usage during the night is generated by inserting a random number (<3) of activations at random hours (0–4 a.m.) during the night.

The aggregate power of the house is adjusted accordingly to the new generated consumption of the kettle resulting in an energy dataset annotated with abnormal usage. This new data is used as input to evaluate the activity monitoring module. From the first evaluation phase, the pre-trained model is used to identify the consumption of the kettle during the monitoring period. Before evaluating the activity monitoring, the authors argue that it is necessary to re-evaluate the disaggregation error to assess if the abnormal activations cause a deterioration in the disaggregation error. The evaluation of the activity monitoring module is then performed using both the actual consumption of the kettle and the predicted consumption obtained with a NILM model. The goal from using both data sources is: (1) To evaluate the performance of the monitoring module, and (2) to assess the influence of the disaggregation error on the monitoring module.

### 4.2. Data Description

All reported results during the experimental setup conducted in the current study were obtained using the REFIT [39] dataset. This dataset contains power consumption from 20 households for two years in the UK, with a sampling rate of 8s. We chose two households from this dataset. We justify the choice of these two households by the fact that they record real energy consumption for senior adults living alone (https://reshare.ukdataservice.ac.uk/852367/11/Documentation.zip, accessed on 30 December 2021), illustrated in Table 4, which represent a perfect fit for our framework. The first house gathers energy consumption from a retired couple over approximately two years. The second house records the energy data of a retired female for an approximate period of one year.

**Figure 4:**
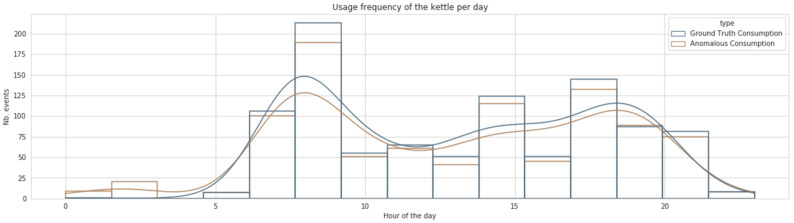
The daily consumption profile of house 4.

Figure 4 highlights the daily consumption of the kettle over 24 h for the whole considered period (see Table 5). It contains two different histograms representing the data before and after inserting the abnormal usage patterns. These anomalous daily patterns were inserted as described in Section 4.1. The occupants from house 4 possess typical behaviour where the kettle is used from 5 a.m. to 10 p.m. These occupants use the kettle extensively in the morning and the evening and exhibit moderate usage during the middle of the day. During the nights of the studied period, the kettle has never been used(see blue curve in Figure 4). As shown, the rate of anomalous days was inserted in a controlled manner and only slightly altered the original consumption curve (shown in blue). This house represents a good example of a scenario where the observation phase captures only typical behaviour.

**Figure 5:**
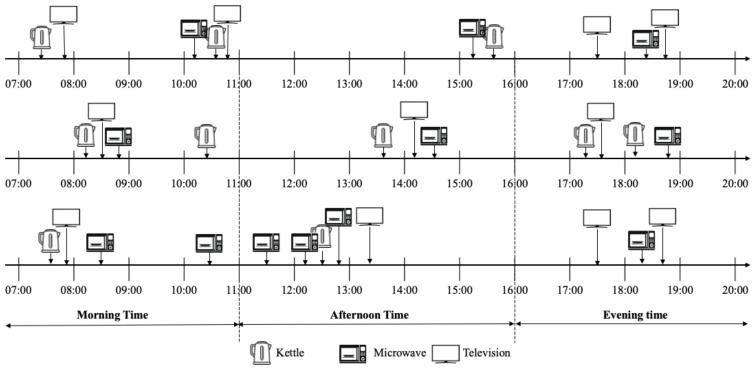
The daily activities of house 4 from 11 October 2013 to 13 October 2013.

To provide a more concrete understanding of the daily routines in house 4, Figure 5 presents a detailed illustration of all the appliances used during three consecutive days (from 11 October 2013 to 13 October 2013). The figure shows strong evidence for the representativeness of the information summarised in the consumption profile. As observable in Figure 5, the cooking activity for house 4 is mainly performed twice a day during the morning time (from 7 a.m. to 11 a.m.), with some variations in the time represented by the first peak in the curves from Figure 4. Similar observations can be deduced for the afternoon and the evening times.

**Figure 6:**
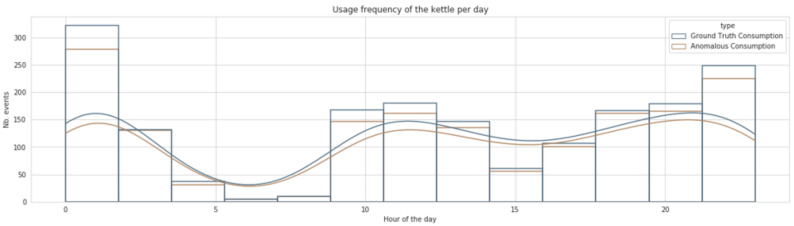
The daily consumption profile of house 11.

On the other hand, Figure 6 illustrates the daily consumption profile of house 11. For this house, the kettle usage is relatively moderate during the day, with more relevant usage in late hours (from 6 p.m. to 10 p.m.). The analysis of daily consumption from house 11 revealed that the occupant already has an unusual consumption pattern where extensive usage of the kettle is remarkable during the night and seems to be part of the subject’s daily routine as provided in the original dataset. In the case of this house, we annotate the existing data relative to usage during the night and data gaps included in the dataset and only introduce synthetic daily usage that is divergent from the usual pattern of the occupant. The data from this house would provide a good example of the sensitivity of the proposed framework to the observation phase as it would also integrate unhealthy usage. It is expected that the resulting data for this household would not allow the detection of the kettle’s usage during the night as it is part of the typical routine of the householder. This second use case will highlight the extent to which the decision about the abnormality remains relative to the occupant’s routines and the data recorded during the observation phase. Therefore, it will serve as a good demonstrative example of the importance of relativity in differentiating between abnormal and normal behaviour.

## 5. Results

### 5.1. NILM Evaluation

Table 6 illustrates the disaggregation results for the kettle considering the four metrics. The table summarises the results for both houses representing the seen and unseen scenario as the models were trained considering only data from house 4. The best obtained results for each metric are highlighted in bold.

As seen from Table 6, the four considered deep models outperform the classical models (CO and FHMM), considering all metrics except for the recall. These observations translate to the fact that these classical models perform well at identifying the real ON events (small number of FN) however tend to confuse the use of other appliances with the kettle (big number of FP) in both scenarios. On the other hand, the considered deep models provided decent performance where we recorded a minimum f1-measure of 77%. The Seq2Seq and Seq2Point baselines provided the best results considering classification performance metrics where they yielded an f1-measure greater than 85%. Nonetheless, a closer inspection of the precision and the recall reveals that the rate of overestimated ON states (FP) is lower in the case of Seq2Point, while the rate of undetected ON states (FN) is lower in the case of Seq2Point. While providing a lower MAE, the Temp-Pooling model demonstrated also a lower f1-measure than both previous baselines. This observation, while preliminary, suggests that this model is more conservative in predicting that the kettle is ON. Finally, the UNET model yielded very competitive results considering the MAE with an f1-measure that approximated the two considered baselines. It reduced the MAE with a factor of 2 compared to the Seq2point while providing only a 5% deterioration in the f1-measure compared to the best obtained value. We argue that this model provides a good trade-off between the undetected ON states (FN) and the overestimated ON states (FP), as well as the power consumption values.

**Figure 7:**
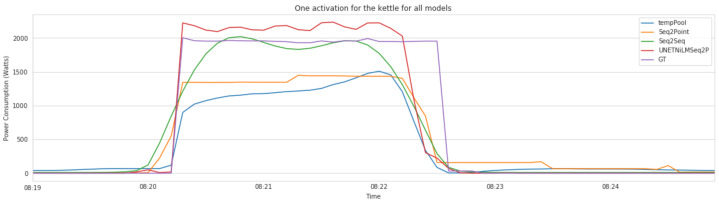
A morning activation of kettle for 1 January 2015 from house 4.

Figure 7 illustrates one activation of the kettle during the morning of 1 January 2015 from house 4. It illustrates the predictions generated by the four deep models and the real consumption (GT). The figure shows that indeed the four models succeed in detecting the kettle’s usage. It can be noticed that there is a slight delay of a few seconds between the predictions and the actual data. Nonetheless, we argue that this delay is negligible and will have a very small to no effect on activity monitoring performed on an hourly basis. The figure shows that the UNET outperforms the other models where it generated an activation that is very similar (shown in red) to the actual activation (shown in purple). Considering this observation and the results obtained in Table 6, we argue that the UNET model is particularly relevant in real scenarios where a single network would be used for both activity monitoring and energy estimation. The activity monitoring can thus be offered as an extra service that relies on the disaggregation output.

In the case of the unseen scenario (house 11), a deterioration in the performance can be noticed for all models, mainly caused by an increased number of undetected ON events as recorded per the recall. A possible justification can be related to the different operational characteristics of the kettle in house 11. Remarkably, the Temp-Pooling model demonstrated a significant draw in the performance with an MAE multiplied by a factor of 2.7 and a difference of 23% in the f1-measure. It could be the case that this model over-fitted the data from house 4 and cannot generalise to data from unseen houses. The three remaining deep models yielded acceptable results with a minimum f1-measure of 71%. The UNET model also demonstrated the best trade-off considering both the MAE and f1-measure.

In summary, NILM approaches provided acceptable results. Deep models demonstrated outstanding improvements in comparison with classical approaches. In particular, the UNET model yielded good performance even on the unseen scenario where it yielded the lowest deterioration in the f1-measure. Thus, we argue that this model is the most suitable for activity monitoring. Therefore, only the UNET model will be considered during the rest of the experimental setup, where the pre-trained model will directly be used to generate the predictions for the activity monitoring module.

Table 7 illustrates the re-evaluation results of the pre-trained UNET model for the considered period (see Table 5) in the case of both houses that will serve as input to the monitoring process. As the newly generated data contains new randomly generated patterns, it is expected to notice a deterioration in the performance. The goal of the authors is to assess the new error and evaluate the ability of the UNET model to generalise to the new consumption patterns. As the table demonstrates, the UNET still provides decent performance in both seen and unseen cases. A decrease of 8% in the f1-measure is recorded in the case of house 4 due to a lower recall value (85% with real data vs. 73% with the newly generated data), translating into a higher rate of undetected ON events for the kettle (FN) in this case. The same observation can be made for the f1-measure in the case of house 11. Nonetheless, the decrease in house 11 is caused by an overestimation of ON events (FP). These findings, while preliminary, suggest that UNET is capable of decently identifying the new activations with anomalous usage time. Nonetheless, a more advanced training protocol considering the potential of anomaly occurrence would allow to mitigate the recorded deterioration in the performance as previously suggested in related work [48]. The authors admit that this aspect remains challenging to design but must be addressed in future work to unlock the full potential of applying NILM in activity monitoring.

### 5.2. Activity Monitoring and Anomaly Detection

The current section presents the results of the monitoring module considering the data from houses 4 and 11 as described in Section 4.1. Table 5 illustrates the periods for both observation (building the consumer profile) and monitoring. For house 4, a period of 51 days is used as the observation phase to capture the behavior of the retired couple. The consumption of the subjects from the same house is monitored for a period of more than 300 days. Similar periods are fixed for house 11 with respect to data availability.

The results obtained from the activity monitoring module relative to the identification of anomalous days, considering both the actual consumption and the consumption generated by the UNET model, are illustrated in Table 8. The table summarises the results of the detected anomalous days without distinguishing between the causes of the anomaly (the pre-defined anomalous classes). As the introduced anomalous daily consumption only considered the usage time, only the activity level anomaly (activity curves) was enabled during the current evaluation. More precisely, the appliance level anomaly (using similarity matrices) is more relevant when considering complex scenarios with multiple features (day of the week, day of the month, the season, operational characteristics, etc.) for each appliance translating into different interpretations. Specific control rules on the usage time of the identified anomalous days remain a better alternative for providing interpretations when considering only the usage time, resulting in less computational resources.

**Figure 8:**
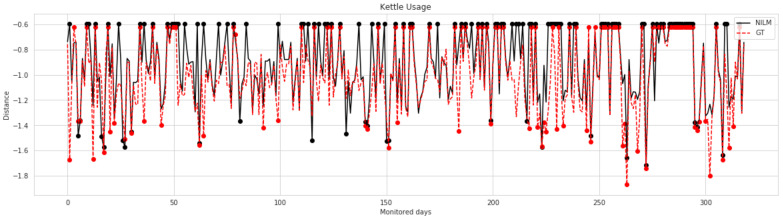
The Jensen–Shannon Divergence (JSD) in the case of house 4.

In the case of house 4, both input sources, the UNET predictions, and the real consumption yielded acceptable results with a minimum f1-measure of 67%. Both data sources provided precision values higher than the recall values. The previous observations imply that in both cases, the number of ordinary days identified as anomalous (FP) is greater than the number of undetected anomalous days (FN). A viable justification for this finding can be that the dataset already contained anomalous patterns that were not considered during pre-processing. When comparing the results generated by both inputs, we record a difference of 10% in the f1-measure. A closer look at the precision and recall values reveals that both data sources have approximate precision values but a difference of 15% in the recall values. The previous observation is directly linked to the results obtained in Table 7 where we noticed an increase in the rate of undetected ON states after the introduction of abnormal usage, which would directly lead to a higher rate of undetected abnormal days that are related to those activations. To further evaluate the influence of the first module on activity monitoring, Figure 8 details the distance calculated for each day from the monitored period where the curve in red represents the distance when considering the actual power consumption and the curve in black represents the distance when considering the data generated with the UNET model. The days that were identified as anomalous are highlighted with bold dots. As shown, both generated curves exhibit the same overall shape with minor differences where the module either failed at classifying the day as anomalous or misidentified it as anomalous.

As expected, the activity monitoring module could not detect the days labeled as anomalous (mainly days with usage during the night) during the pre-processing, where it yielded a very low f1-measure with both input types. These results are due to the extensive usage of the kettle that was labelled during pre-processing as anomalous but considered normal by the proposed framework as it was part of the observed data. The case of this house can be viewed from two different perspectives. The first is the case where the night usage is considered anomalous. In this case, if the observed data is not validated, the proposed framework will fail to identify what is hypothesised to be abnormal (i.e., usage of hand-operated appliances during the night). This finding highlights a central failing point for our contribution where it shows its sensitivity to the data recorded during the observation phase that needs to be established in a very controlled manner and under the validation of professionals. The second perspective would be the case where the kettle (or other hand-operated appliances) usage during the night is just part of the subject’s routines and is far from being an indicator of a sleep disorder. This would mean that what is anomalous for one individual could be part of a routine for another individual. This finding stresses the importance of the observation phase to define custom anomalies. In this case, the definition of abnormal behavior remains relative.

## 6. Discussion

The manuscript at hand proposed a new activity monitoring framework using NILM and suggested an evaluation protocol that considers different modules of the framework. Accordingly, the evaluation results, on two case studies from a publicly available dataset, were presented to simulate a realistic scenario.

The evaluation of the first module relative to the load disaggregation using both classical and deep approaches revealed that NILM models provide good results in identifying the kettle’s activations and power consumption. Notably, deep models provided the best performance for this module. Compared to classical models, deep baselines demonstrated their competitiveness, confirming findings from recent literature [32]. Even with the unseen scenario, these models yielded acceptable performance. This finding provides encouraging evidence to transfer learning from available houses not relative to adults living alone. Moreover, advanced models, such as the UNET, demonstrated very competitive performance in estimating power consumption. The authors argue that the UNET model is particularly interesting since it performs both power and state predictions (multi-task model) with uncertainty estimation. Unfortunately, in its current version, the proposed framework does not benefit from the uncertainty generated by the model. Nonetheless, the authors plan to include it in future versions.

The evaluation of the second component relative to the activity monitoring also yielded acceptable performance. The activity curves demonstrated good representativeness of the overall activity patterns. Nevertheless, we argue that they are more suitable for activities that rely on frequently used appliances during a single day. We stress that it could be the case that they are less effective in activities inferred based on less frequently used appliances, such as the washing machine. A more elaborated version of the proposed framework would rely on hierarchical activity curves, including other temporal dimensions such as the day of the week. Adapting established methods for activity monitoring or combining them demonstrated good results but remains subject to future improvements. The activity monitoring of older adults remains a sensitive health care service that requires more robust and elaborated models to help mitigate the number of errors. Moreover, using two case studies demonstrated that the definition of anomalous behaviour remains difficult and dependent on the context.

On the other hand, the evaluation of the activity monitoring module considering both real data and NILM predictions allowed to assess the propagated error that was estimated to be around 10% for the f1-measure. This difference shows encouraging results for using deep NILM models in activity monitoring and demonstrates that more elaborated models (both NILM and monitoring models) would help to mitigate this error. The presented case studies also highlighted the sensitivity of this module to the data recorded during the observation phase.

The proposed framework showed potential for benefiting from the new electrical grid in active and assisted living. To the best of the authors’ knowledge, this is the first application of deep NILM models in activity monitoring with an explicit assessment of their effect on the activity monitoring algorithms. Nevertheless, a source of limitation in the proposed solution is its high dependency on the data recorded during the observation phase that must represent the average occupant’s routine. Moreover, the scope of this study was limited in terms of the set of monitored activities. The authors intend to consider a more extensive set of activities with more appliances in future work. Furthermore, we argue that gaining a deeper understanding of the challenges imposed by the problem can only be enabled with more established datasets. More particularly, a significant obstacle in evaluating the applicability of NILM in activity monitoring is the non-availability of annotated data. The authors overcame this problem by introducing abnormal synthetic usage and annotating available suspicious usage time. The generated data allowed the evaluation of the proposed framework on data from two different houses. However, this aspect remains the main limitation for the evaluation protocol adopted in the experimental setup and a major obstacle towards the generalizability of these results. We urge the need to establish more elaborated datasets to develop more complex scenarios and more extensive evaluations of activity monitoring approaches based on NILM.

## 7. Conclusions

Adopting Non-intrusive Load Monitoring for daily activity monitoring is a promising approach. The current manuscript proposed a new framework for activity monitoring based on this paradigm. The evaluation of the whole modules of the framework revealed that deep NILM models are a good fit for the problem with an influence of –10% in the f1-measure of the activity monitoring algorithm. Nonetheless, the generalisability of these results is subject to certain limitations. For instance, the data used during the evaluation remains limited regarding the number of houses and appliances. Further research may focus on providing larger energy datasets of older adults, allowing deeper evaluations. Despite its limitations, the current study is the first to suggest using deep NILM models for activity monitoring and to measure the error propagated from the NILM algorithm on activity monitoring. In this regard, the presented work can be considered as a proof of concept that the basic algorithms are appropriate. The plan for future activities is to transfer the method from artificial environments into real-world settings, similar to the ones described in [8], which is the past work of one of the authors. In this paper, the authors illustrate the possibilities and limitations of a conventional AAL approach, i.e., one based on the installation of additional components to observe the activity. Due to the spread of smart meters, evaluating the solutions presented in this paper in real-world settings on a larger scale should be feasible and is planned for our future work.

## Figures and Tables

**Table 1:** An example of instrumental daily activities and related appliances.

Activity	Indicating Appliances
Cooking	Oven, kettle, coffee maker, microwave, toaster
Ironing	Iron
Entertaining	Television, audio system
Laundry	Washing machine, washer dryer
Cleaning	Dishwasher, vacuum cleaner
Sleeping	All hand operated are off during night

**Table 2:** Recent activity monitoring approaches based on energy data.

Approach	Use of a NILM Technique	Type of Data	Data Available	Model Used for Activity Monitoring
[37]	**✗**	Lab experiments	**✗**	NA *
[36]	**✗**	Lab experiments	**✗**	NA *
[6]	**✗**	Lab experiments	**✗**	Bayesian Machine Classifier
[14]	**✗**	Lab experiments	**✗**	Statistical model
[7]	**✗**	Lab experiments	**✗**	HMM combined with log Gaussian Cox process
[16]	**✗**	Lab experiments	**✗**	The use of a pre-defined On/Off database
[4]	**✗**	The HES energy dataset	**✓**	Dempester Shafer Theory (DST)

NILM: Non-Intrusive Load Monitoring; HES: Household Electricity Survey; HMM: Hidden Markov Model; * NA: Description not available.

**Table 3:** The details of the data used during phase 01.

House	Training Period		Testing Period	
	Start	End	Start	End
4	1 April 2014	30 July 2017	1 May 2015	30 May 2015
11	-	-	1 October 2014	28 October 2014

**Table 4:** The meta-data of house 4 and 11 from the REFIT dataset.

House	Pseudonyms	Age Band	Occupation	Start of the Measurement	The end of the Measurement	Period’s Length (Days)
4	Henry	55–64	Retired	13 October 2013	7 January 2015	635
	Louise	55–64	Retired			
11	Sarah	65–74	Retired	6 June 2014	30 June 2015	393

**Table 5:** The periods of time used during phase 02.

House	Initial Observation Period		Monitoring Period	
	Start	End	Start	End
4	1 September 2014	21 October 2014	22 October 2014	30 September 2015
11	1 November 2014	21 December 2014	22 December 2014	16 August 2015

**Table 6:** The results of the disaggregation performance.

	House 4 (Seen Scenario)				House 11 (Unseen Scenario)			
	MAE	F1	Precision	Recall	MAE	F1	Precision	Recall
CO	232.9	0.26	0.15	0.97	279.5	0.23	0.13	0.91
HMM	67.5	0.18	0.10	0.97	170.7	0.18	0.09	0.93
Seq2Point	9.6	0.85	**0.89**	0.81	17.5	0.71	0.79	0.65
Seq2Seq	14.0	**0.89**	0.86	**0.91**	25.5	0.74	0.85	**0.66**
Temp-Pool	7.3	0.77	0.85	0.69	20.3	0.54	**0.93**	0.37
UNET	**4.4**	0.83	0.82	0.85	**12.5**	**0.75**	0.91	0.64

**Table 7:** The results of the re-evaluation of the UNET model.

	House 4				House 11			
	MAE	F1	Precision	Recall	MAE	F1	Precision	Recall
Real data	4.4	0.83	0.82	0.85	12.5	0.75	0.91	0.64
Augmented data	5.4	0.77	0.82	0.73	23.9	0.63	0.64	0.61

**Table 8:** The results of the evaluation of the activity monitoring module.

Input Source	House 4			House 11		
	F1	Precision	Recall	F1	Precision	Recall
UNET predictions	0.67	0.63	0.71	0.03	0.8	0.01
True consumption	0.77	0.69	0.86	0.007	1.0	0.003

## Data Availability

Publicly available datasets were analyzed in this study. This data can be found here: https://www.refitsmarthomes.org/datasets/, accessed on 25 December 2021.
